# Molecular Dynamics of a Polymer Blend Model on a Solid Substrate

**DOI:** 10.3390/molecules30081734

**Published:** 2025-04-12

**Authors:** O. E. Ayo-Ojo, M. Tsige, G. T. Mola, A. Rotondo, G. L. La Torre, G. Pellicane

**Affiliations:** 1 School of Chemistry & Physics, University of KwaZulu-Natal, Pietermaritzburg, Private Bag X01, Scottsville 3209, South Africa; oluwatumininuayoojo@gmail.com (O.E.A.-O.); mola@ukzn.ac.za (G.T.M.); 2 Department of Polymer Science, University of Akron, Akron, OH 44325, USA; mtsige@uakron.edu; 3 Dipartimento di Scienze Biomediche, Odontoiatriche e delle Immagini Morfologiche e Funzionali, Università degli Studi di Messina, I-98125 Messina, Italy; archimede.rotondo@unime.it; 4 National Institute of Theoretical and Computational Sciences (NIThECS), Pietermaritzburg 3209, South Africa

**Keywords:** interface, polymer blend, adsorption, molecular dynamics

## Abstract

We performed extensive molecular dynamics simulations using a bead–spring model to investigate the interfacial behavior of blends of linear and cyclic polymer chains confined between two planar, attractive substrates. The model system was studied over a range of chain lengths spanning an order of magnitude in the number of beads for varying blend compositions and for two different levels of substrate affinity. For short chains, we observed the preferential adsorption of linear chains at the substrate interface when they are the majority component (10% cyclic chains) as well as at equimolar composition. In contrast, for longer chains, cyclic chains are preferentially enriched at the interface. These results extend recent findings from neutron reflectivity experiments—where the enrichment of cyclic polystyrene chains at low-energy surfaces was demonstrated—to systems under solid confinement, providing deeper insight into the structural behavior of topologically distinct polymers near interfaces. This work highlights the potential for tuning interfacial composition and properties in polymer blends through topological design, with implications for advanced coatings, membranes, and nanostructured materials.

## 1. Introduction

Polymer chemistry and physics are important to adhesion because modern adhesives are engineered with polymeric materials [1]. In fact, synthetic polymers are used to produce adhesives and coatings for microelectronics, tissue engineering, regenerative medicine, and 3D printing [2]. The lightness and resistance to fatigue of polymer adhesives is also exploited in aerospace and automotive engineering [3].

In structural adhesives, which are used to bind metals and composites, adhesion can be triggered by chemical bonding so as to establish strong attractive interfacial interactions. However, in thermoplastic adhesives, the system is initially in the (“hot”) melt state and strengthens upon solidification. The interactions occurring within the top 3–5 molecular layers and changes in polymer conformation at the interfaces play a crucial role in establishing strong and durable coatings of these polymer melts [4].

Thin polymer films are used in several technological applications and the influence of confinement on the flexible conformations of the chains is a subject of wide interest [5,6]. In fact, adhesion is clearly influenced by the structural features of the interfacial layer of the polymer melt. When the polymer melt is a blend of two distinct polymers, its interfacial density profile can be tuned by the different chemical properties and molecular weights [7] of the two molecular species and the different attractive forces they experience in proximity to the substrate.

However, when the monomer chemistry and molecular weight of the two polymers are similar, but their chain architectures are different, the surface structure can also be altered with respect to the bulk by selective enrichment of one of the two species at the interface. Choi et al. showed that conformational entropy alone can significantly influence the spatial distribution of polymers at the interface, favoring the enhancement of ring-shaped polymers [8]. By numerically studying mixtures of linear and branched chain molecules, Yethiraj [9] found that if linear chains interact among themselves through steric (hard-sphere) interactions only, they preferentially segregate to the surface; however, when attractive interactions are switched on among linear chains, they become more depleted at the surface than branched chains. Cyclic chains have a peculiar closed-loop structure, which makes them exhibit material properties that differ considerably from those of linear and branched polymers of the same molecular weight [10]. Recently, neutron reflectivity experiments on binary blends of linear and cyclic polystyrene chains provided evidence that the segregation of cyclic polymers depends on the molecular weight of the polymer chains [11]. When chains possess a low molecular weight (≈ 2 k), linear polymers enrich the surface, as was first demonstrated by using surface layer matrix-assisted laser desorption ionization time-of-flight mass spectrometry (SL-MALDI-TOF MS) [12]. However, cyclic chains preferentially absorb onto the surface for relatively large weights (≈ 37 k), and the threshold between the two regimes was conjectured to arise from the competition between poorer packing of cyclic polymers and the smaller conformational entropy loss at the surface [11]. A very useful computational bead-and-spring model for studying polymer adhesion was originally introduced by Kremer and Grest (KG) [13]. While the KG model is a physics-based coarse-grained model, it can be mapped onto a large number of polymers to reproduce some of their large-scale properties in the molten state, such as, for instance, their Kuhn and packing lengths [14]. Molecular dynamics is an efficient computational method to study polymer chain conformations and interchain packing, especially when the polymer matrix is exposed to an inhomogeneous environment, as an interface to a solid substrate or nanoparticles [15]. By performing molecular dynamics (MD) simulations of bead–spring models of free-standing, binary mixtures of linear–cyclic polymer chains, we were able to provide a fundamental understanding of the role of entropic and enthalpic interactions towards the surface excess composition of linear and cyclic polystyrene chains [16,17]. Linear–ring blends of polymer chains are considered as important models to understand the role of chain topology in driving preferential absorption at the interface because recent experimental results have demonstrated the toughness of cross-linked ring–linear polymer blends; an efficient compression method with good numerical accuracy that preserves their topology was also developed for these models [18].

While it is well understood that for a low-energy surface, such as air or a vacuum, the polymer species with the lower cohesive energy density has the lower surface tension and is favored at the interface [19,20], the outcome could be different in the case of a solid substrate because of the presence of adhesion forces. In fact, several phase transitions can influence adsorption when a polymeric system is exposed to a very attractive solid substrate, including pre-wetting, wetting, and layering [21,22,23,24]. Several experimental and computational studies addressed the case of strong substrate attraction; however, for polymer blends with different chain architectures, if the wall is very attractive, the strong affinity to the chain beads could make the enthalpic component of the interfacial free energy predominant to the entropic contribution carried by the chain architecture. Therefore, the study of binary mixtures of linear and cyclic chains in a situation where the strength of substrate attraction and attractive particle–particle interactions are not very different could allow us to better understand the role of chain topology in driving preferential absorption on a solid substrate.

In this paper, we aim to achieve a fundamental understanding of the surface structural features of linear and cyclic chains confined between two planar, mildly attractive, solid substrates. We will perform molecular dynamics (MD) simulations of KG bead–spring models of binary mixtures of linear and cyclic chains at different lengths and compositions and at different levels of affinity to the chain beads to the substrate. Since the experiments reported in the literature for blends of linear and cyclic chains have been conducted in the regime where linear chains are the majority component [11,12], we will mainly consider asymmetric compositions of the two chains. Our focus will be to characterize the structural profile of the blend near the interface and to relate it to the main ingredients of the interfacial free energy, i.e., conformational entropy and energy of the chains.

## 2. Materials and Methods

### 2.1. The Model

The Kremer–Grest bead–spring model [25] is widely recognized for its versatility in molecular dynamics (MD) simulations of polymeric systems. In our study, polymers consist of chains made of sequential monomers with uniform mass (*m*), forming either cyclic (closed-loop) or linear (open-loop) structures through bonded interactions.

The interactions between non-bonded monomers are represented by the truncated and shifted Lennard-Jones (LJ) potential [26] at $r_{c}=2.5\sigma$:

(1) $$E_{\mathrm{LJ}}\left(r\right)=\left\{\begin{array}{cc} 4\epsilon \left[{\left(\frac{\sigma }{r}\right)}^{12}-{\left(\frac{\sigma }{r}\right)}^{6}\right]+\epsilon_{\mathrm{LJ}}, & r\leq r_{c}\\ 0, & r>r_{c}, \end{array}\right.$$

where *r* represents the inter-monomer distance, σ is the distance where the LJ potential becomes zero (also commonly identified with the bead size), ϵ is the depth of the potential well, and $\epsilon_{\mathrm{LJ}}$ ensures the continuity of the potential at $r=r_{c}$ (the truncated and shifted LJ potential becomes zero at $r=r_{c}$).

For bonded monomers, the repulsive part of the LJ potential (shifted at the cut-off distance $r_{c}=2^{1/6}\sigma$) is combined with the finitely extensible nonlinear elastic (FENE) potential [27]:

(2) $$E_{f}\left(r\right)=\left\{\begin{array}{cc} -\frac{1}{2}Kr_{0}^{2}\ln\left[1-{\left(\frac{r}{r_{0}}\right)}^{2}\right], & r\leq r_{0}\\ \infty , & r>r_{0}, \end{array}\right.$$

where $r_{0}=1.5\sigma$ is the maximum bond extension, and $K=30\epsilon /\sigma^{2}$. We applied no angle potential among three consecutive beads.

The beads of any chain interact with a flat wall perpendicular to the z-direction of the simulation box via the LJ 9-3 potential:

(3) $$E_{\mathrm{LJ}\;9\text{-}3}\left(z\right)=\left\{\begin{array}{cc} \epsilon_{w}\left[\frac{2}{15}{\left(\frac{\sigma }{z}\right)}^{9}-{\left(\frac{\sigma }{z}\right)}^{3}\right]+\epsilon_{\mathrm{LJ}9-3}, & z\leq z_{c}\\ 0, & z>z_{c}, \end{array}\right.$$

where *z* is the distance of the beads from the wall and $z_{c}$ is the distance at which the bead and the wall will no longer interact; we considered two different values for the energy parameter $\varepsilon_{w}$, $\varepsilon_{w}=\varepsilon =1$ or $\varepsilon_{w}=2$. The energy of the wall potential is shifted by $\epsilon_{\mathrm{LJ}9\text{-}3}$ so that the wall–bead interaction is zero at the cut-off distance $z_{c}=2.2\sigma$. We modeled the substrate–fluid interactions with the LJ 9-3 potential instead of using the more sophisticated Steele potential [28] because LJ 9-3 has a simple analytical form and does not need to select values for the adjustable parameters of the Steele potential to resemble a specific wall–fluid interaction.

Both Brownian and frictional forces influence monomer dynamics [29]; in fact, the total external force acting on the beads is given by the following:

(4) $$F=F_{f}+F_{B}+F_{E}$$

where $F_{f}$, $F_{B}$, and $F_{E}$ are the frictional drag force, Brownian force, and effective spring force (given by the FENE potential), respectively. The frictional drag and the Brownian forces are used to mimic the presence of solvent in terms of viscous damping and the frequent random collisions between the bead and the solvent molecules at a particular temperature *T*. The frictional drag is expressed as follows:

(5) $$F_{f}=-\left(\frac{m}{\zeta }\right)V$$

where *V* is the velocity of the bead, ζ is the damping factor, and *m* is the mass of the bead. The Brownian force $F_{B}$ is used to represent the frequent random collisions between the bead [30] and the implicit solvent molecules at a particular temperature *T*. According to the fluctuation–dissipation theorem, its magnitude is given by the following:

(6) $$F_{B}=\sqrt{\frac{k_{B}Tm}{\zeta \Delta t}}$$

where $k_{B}$ is the Boltzmann’s constant, $T=\epsilon /k_{B}$ is the temperature, *m* the mass of the bead, and Δt is the timestep size.

### 2.2. Simulation Details

We generated the initial configurations of mixtures of linear (open) and cyclic (loop) chains with $N_{b}=10,40,\;\mathrm{and}\;100$ beads per chain in the simulation box. The bulk composition of cyclic chains is defined as $C_{0}=N_{c}^{0}/\left(N_{c}^{0}+N_{l}^{0}\right)$, where $N=N_{c}^{0}+N_{l}^{0}$ represents the total number of cyclic and linear chains, respectively. We considered a number of chains of each species, ranging from 20,000 (10-mers) down to 2000 (100-mers), corresponding to a total number of beads $N=2\times 10^{5}$. The simulations were performed using the LAMMPS package [31], employing a velocity Verlet algorithm and a Langevin thermostat to stabilize the temperature. We used the Lennard-Jones parameters σ and ϵ as units of length and energy. All the physical quantities homogeneous to a length and a energy, which are reported in the following part of the manuscript, will be always assumed to be in units of σ and ϵ, unless otherwise specified (examples: radius of gyration, energy per beads). The temperature T, the damping constant of the Langevin thermostat ζ, and the timestep Δt were set, respectively, to $T=\epsilon /k_{B}\;=1$, $\zeta =2\tau^{-1}$, and Δt=0.005τ, where $\tau ={\left(m\sigma^{2}/\epsilon \right)}^{1/2}$ is the time unit in reduced LJ units.

The initial configuration was prepared by randomly inserting the centers of mass of linear and cyclic polymers onto the sites of a parallepiped lattice. The lattice parameters of the parallepiped box were chosen to be large enough to avoid the formation of knots between cyclic and linear polymers. Then, a LAMMPS MD simulation with the directive fix/DEFORM was used to shrink the box isotropically to the desired volume. These initial configurations were first equilibrated in the NPT ensemble at pressure P = 0 and T = 1.0 using both a Langevin thermostat and a Berendsen barostat with periodic boundary conditions in all three directions for more than 50,000τ, which well exceeds the relaxation time of the end-to-end vector autocorrelation function for the longest chains of about 24,000τ. Then, the periodicity along the z-direction was removed and the chains crossing the boundaries regenerated. Finally, the flat bottom wall was placed at a distance from the bead possessing the lowest z coordinate equal to the distance corresponding to the minimum energy of the LJ 9-3 potential; similarly, the z-position of the top flat wall was chosen at a distance from the bead, possessing the highest z coordinate corresponding to the minimum energy of the LJ 9-3 potential. NVT simulations using a Langevin thermostat were then run for 300,000τ and quantities of interest were averaged over the last 100,000τ. We verified that the average density in the middle of the films after equilibration was the same as the bulk density (within the statistical error) at the end of the NPT equilibration runs (see Table 1)).

**Figure 1:**
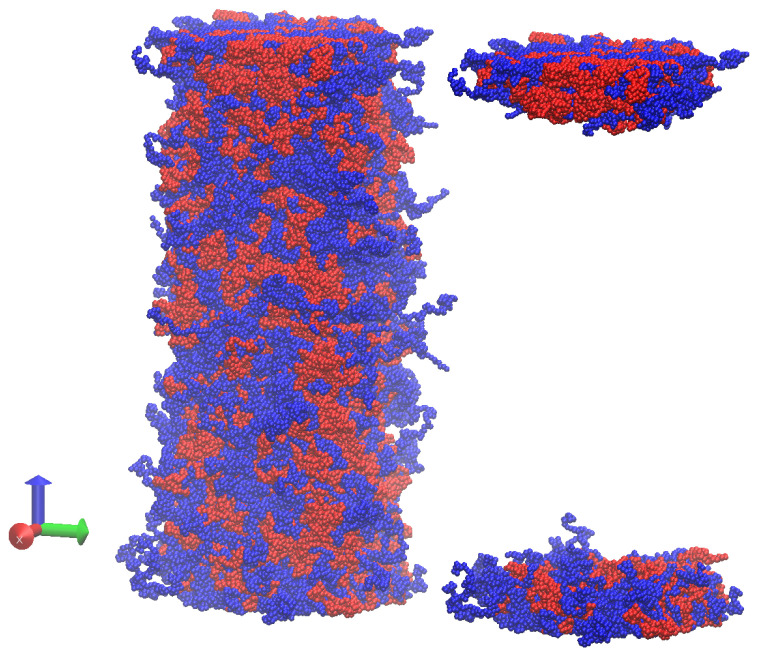
Snapshots of the simulation box for 100 mers at $C_{0}=50\%$. **Left panel**: 2000 chains of cyclic (red beads) and linear (blue beads) chains. **Right panel**: only chains with beads closer than 12σ to the walls are reported.

In Figure 1, we show a snapshot of the simulation box for an equimolar mixture of 100 m to provide a visual illustration of the typical distances from the substrate(s), where we focus most of our analysis of the interfacial properties. All (average) structural quantities were reported as a function of the distance from the two walls at low and high z values.

## 3. Resultsand Discussion

### 3.1. Density and Local Composition

Before considering the binary mixture with the two different polymer chains, let us try to understand if the sole topology of each chain has an intrinsic influence on its adsorption properties at the wall. Initially, we will consider pure systems consisting of either cyclic or linear chains. In the following discussion, when we look at the profile of any physical quantity as a function of the distance from the wall, our comments will always be intended for the range of distances within three 3D bulk radii of gyration from the interface (whose values are reported in Table 2), unless otherwise specified. We refer to that distance range from the solid substrate as the *interfacial thickness of interest* (ITI). The relatively weak level of wall affinity that we are considering in our model to understand entropic effects driven by the chain topology is particularly relevant to the case of polymer nanometer films. In experiments conducted on such substrates, non-adsorbed chains are washed away by soaking the sample in a good solvent, and the resulting, extremely thin, irreversibly adsorbed layer is found to scale with the radius of gyration $R_{g}$ of polymer chains. For example, the thickness of bound layers of polysterene (PS) polymers onto hydrogen-passivated silicon oxides of the type PS/Si or PS/$SiO_{x}-Si$ varies from nearly half [32] to a bit less than one $R_{g}$ [33]. The surface dynamics is totally suppressed for a thickness of less than one Rg and, overall, can be altered up to thicknesses of $4R_{g}$ [34].

**Figure 2:**
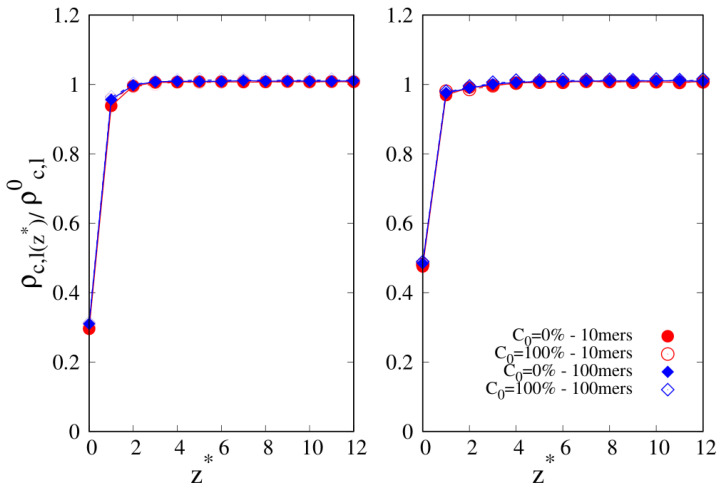
Reduced density profile as a function of the reduced distance $z^{*}=\frac{z}{\sigma }$ from the substrate. Circles: 10-mers; diamonds: 100-mers. Open symbols: cyclic chains ($C_{0}=100\%$); full symbols: linear chains ($C_{0}=0\%$). **Left panel**: wall–bead energy parameter $\epsilon_{w}=1$. **Right panel**: wall–bead energy parameter $\epsilon_{w}=2$. Lines are a guide.

In Figure 2, we focus on 10-mers (ITI: $z^{*}=\frac{z}{\sigma }≲$4 since $R_{g,b}^{\mathrm{cyc}}\approx 1.1$ and $R_{g,b}^{\mathrm{lin}}\approx 1.5$) and 100-mers ($z^{*}=\frac{z}{\sigma }≲$ 15 since $R_{g,b}^{\mathrm{cyc}}\approx 3.5$ and $R_{g,b}^{\mathrm{lin}}\approx 5.2$). Since the affinity of the solid substrate to the polymer species can be varied by, for example, changing the nature of the substrate [35], we also tried to understand the effect of changing the energy parameter $\varepsilon_{w}$ controlling the intensity of blend–wall attraction and making it double, i.e., $\varepsilon_{w}=2$.

The reduced density profiles are defined as $\rho_{i}\left(z^{*}\right)/\rho_{i}^{0}$, where

(7) $$\rho_{i}=\frac{N_{i}\left(z^{*}\right)}{V_{\mathrm{shell}}}$$

and

(8) $$\rho_{i}^{0}=\frac{N_{i}^{0}}{V}.$$

The density $\rho_{i}^{0}$ is the bulk density of the species labeled as i≡{c,l} (c stands for “cyclic” and l for “linear”), where $N_{i}^{0}$ is the number of beads of species *i* contained in the simulation box of volume V. $N_{i}\left(z^{*}\right)$ represents the number of cyclic or linear beads contained in a parallelepiped shell of volume $V_{\mathrm{shell}}=L_{x}L_{y}\sigma$ localized at distance $z^{*}=z/\sigma$ from the wall (with the notation that $z^{*}=0$ representing the parallelepiped shell starting from the wall), where $L_{x}$ and $L_{y}$ are the simulation box lengths along the *x* and *y* directions.

The reduced density profiles are reported in Figure 2 for the blend–wall energy parameter $\varepsilon_{w}=1$ (left panel) and $\varepsilon_{w}=2$ (right panel), where circles are related to 10-mers and diamonds to 100-mers; open symbols are for cyclic chains ($C_{0}=100\%$) and full symbols for linear chains ($C_{0}=0\%$). The symbols are almost overlapped even nearby the interface, regardless of the chain type and length, suggesting that the entropic component to the interfacial free energy due to distinct chain topologies is not sufficient to generate discernible variations in the adsorption at the interface. Of course, the density profiles nearby the interface for $\varepsilon_{w}=2$ (right panel) show an enhanced density by more than 60% compared to $\varepsilon_{w}=1$ (left panel), bringing the density at the interface from nearly 30% of the bulk value for $\varepsilon_{w}=1$ to 50% for $\varepsilon_{w}=2$. As expected, the density nearby the interface for $\varepsilon_{w}=2$ (right panel) is enhanced by more than 60% compared to the case for $\varepsilon_{w}=1$ (left panel), which corresponds to the density changing from nearly 30% ($\varepsilon_{w}=1$) to 50% ($\varepsilon_{w}=2$) of the bulk value.

Let us analyze the results for the reduced density of the blend, starting from the shortest chains considered in this work, i.e., 10-mers. We only analyze the density of the majority species for the cases $C_{0}=10\%$ and 90%, because in our simulations, we fixed the total number of beads (N = $2\times 10^{5}$ beads); furthermore, when we especially consider longer chains, the reduced number of chains of the minority species inside the simulation box can have its local density affected by a large statistical error [36].

**Figure 3:**
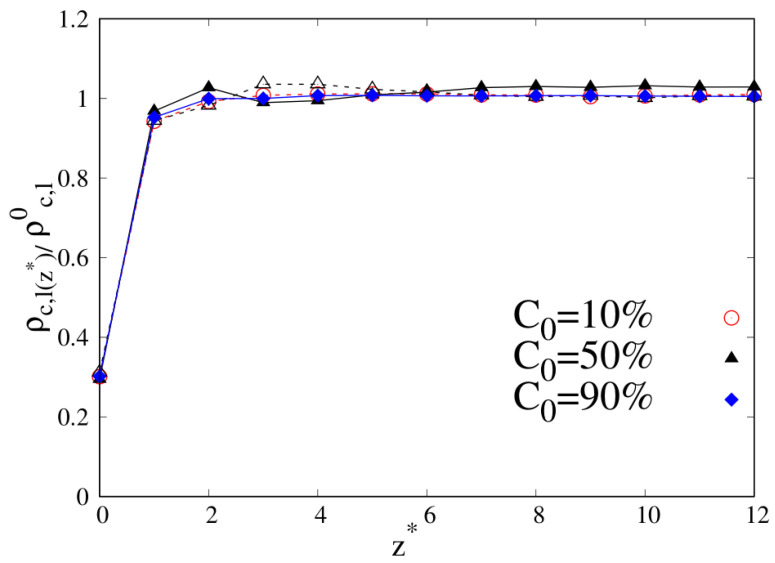
Reduced density profile as a function of the reduced distance from the substrate for 10-mers, wall–bead energy parameter $\epsilon_{w}=1$. Red open circles with dashed line: linear chains at $C_{0}=10\%$; black open triangles with dashed line: linear chains at $C_{0}=50\%$; black full triangles with full line: cyclic chains at $C_{0}=50\%$ (triangles are both for $C_{0}=50\%$ and in the legend only the full triangle for cyclic chains is reported); blue full diamonds with full line: cyclic chains at $C_{0}=90\%$. Lines are a guide.

If we look at Figure 3 (circle: $C_{0}=10\%$, triangle: $C_{0}=50\%$, diamond: $C_{0}=90\%$; full symbols with full lines and open symbols with dashed lines are, respectively, for cyclic and linear chains) and focus on the ITI, we can appreciate that the only case where we clearly observe a reduced density of linear chains going slightly above one is $C_{0}=10\%$. Though there is no evident density enhancement increase with respect to the bulk (reduced densities are systematically lower than, or of the order of unity), such a tiny effect could be compatible with linear chains becoming more concentrated than cyclic chains at the interface, as we will see later. If the evidence is confirmed, then this would be an already peculiar effect in comparison to the uniform behavior observed in the pure systems (see Figure 2), indicating a nontrivial structural effect in the polymeric system arising from mixing two chains with different topologies.

**Figure 4:**
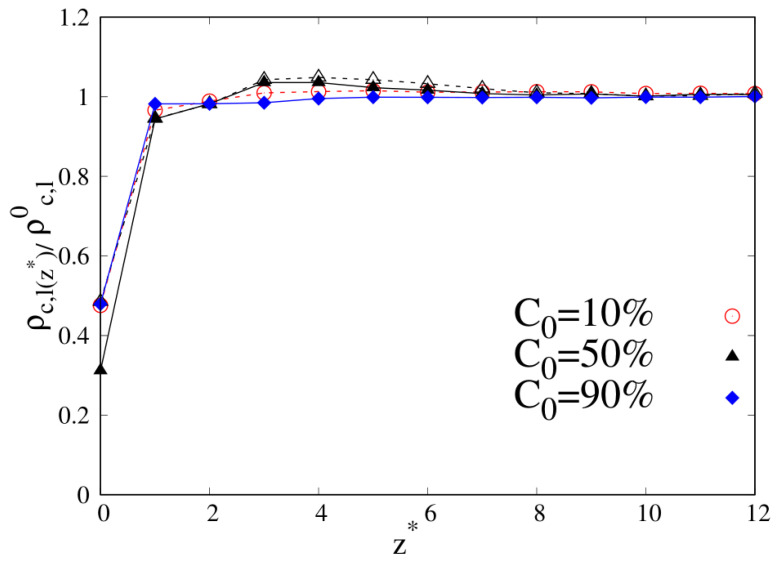
Reduced density profile as a function of the reduced distance from the substrate for 10-mers, wall–bead energy parameter $\epsilon_{w}=2$. Red open circles with dashed line: linear chains at $C_{0}=10\%$; black open triangles with dashed line: linear chains at $C_{0}=50\%$; black full triangles with full line: cyclic chains at $C_{0}=50\%$ (triangles are both for $C_{0}=50\%$ and in the legend only the full triangle for cyclic chains is reported); blue full diamonds with full line: cyclic chains at $C_{0}=90\%$. Lines are a guide.

If we increase the wall attraction to $\varepsilon_{w}=2$ (see Figure 4), we can identify two different ranges of distance from the wall by classifying the ITI into the nearby region (NR) within approximately one $R_{g,b}$ from the wall and the subsequent region (SR) in the interval $R_{g,b}$-$2R_{g,b}$ or $R_{g,b}$-$3R_{g,b}$ from the wall. We clarify that this further classification is only adopted here to discuss the subsequent results more easily. In particular, the NR, which is within one $R_{g}$ from the substrate, has a clear connection to the adsorbed layer [32,33], while the SR (Subsequent Region) is used to analyze the polymer region immediately in contact with the substrate over a few, typical chain length-scales, where the surface dynamics are experimentally observed to be altered with respect to the bulk [34].

We note that the region of depletion of cyclic chains appears more often localized in the SR region when the affinity to the wall is higher ($\varepsilon_{w}=2$) for compositions $C_{0}=10\%$, 50%. A noticeable difference from the case of wall attraction $\varepsilon_{w}=1$ is observed in the NR for the equimolar blend, whose adsorption layer appears more depleted of cyclic chains than before (see the lowest black triangle at $z^{*}=0$).

**Figure 5:**
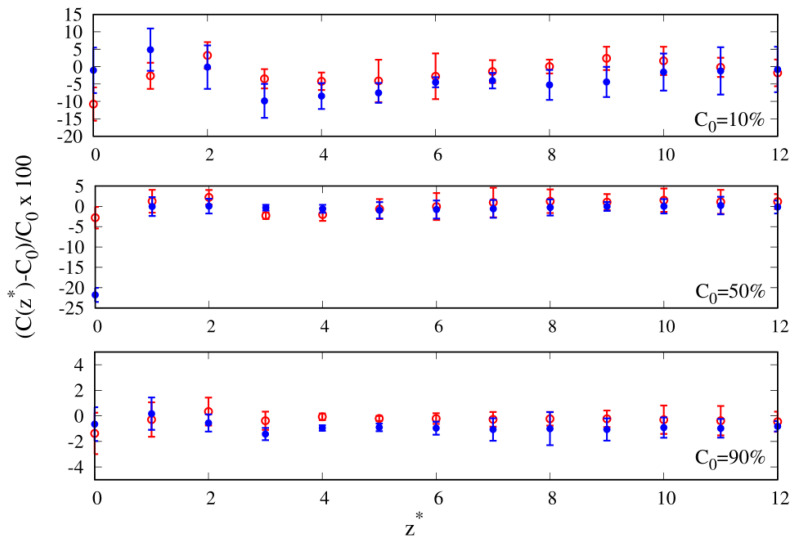
Relative percentage variation in the local composition of cyclic chains as a function of the reduced distance from the substrate for 10-mers and three different compositions (see legend). Red color represents energy parameter of the wall–bead attraction $\epsilon_{w}=1$; blue color represents energy parameter of the wall–bead attraction $\epsilon_{w}=2$.

Density is not the only quantity related to the enhancement of one of the species at the interface. The local composition is also a relevant parameter [37], especially if the composition of the mixture is different from the equimolar one $C_{0}=50\%$. In Figure 5, we report the relative to the bulk value $C_{0}^{b}$ percentage variation in the local composition of cyclic chains

$$C_{0}^{*}=100\times \frac{\left(C_{0}\left(z^{*}\right)-C_{0}^{b}\right)}{C_{0}^{b}},$$

as a function of the distance from the wall, where $C_{0}\left(z^{*}\right)$ is the cyclic composition counterpart of $N_{c}\left(z^{*}\right)$ appearing in Equation (7). In the following discussion, we will focus on those cases where we observe at least a 5% variation with respect to the bulk value.

For $C_{0}=10\%$, we observe that for a mild affinity to the wall ($\varepsilon_{w}=1$), the depletion of cyclic chains is on the order of 10% in the NR region, and for a larger affinity to the wall ($\varepsilon_{w}=2$), it becomes comparable to that figure only in the SR region. Another feature emerging upon the increase in the wall attraction is at the equimolar composition, where we observe an even more depleted cyclic composition of the order of 20% of the bulk composition, confirming what we already noted in the reduced density plot of Figure 4.

Overall, the local compositions confirm the existence of an enhancement of linear chains at the interface, both in the NR and SR regions of the ITI in the regime of lower cyclic compositions $C_{0}$ and up to the equimolar composition $C_{0}=50\%$. These results paint a picture qualitatively similar to the one reported in the literature for free-standing films of linear and cyclic polymer blends of comparable chain lengths, both numerically [16] and experimentally [38]. Our conclusion is that an enhancement of the local composition of linear polymers should be observed experimentally for blends exposed to a solid surface in the case of short chains (10-mers).

**Figure 6:**
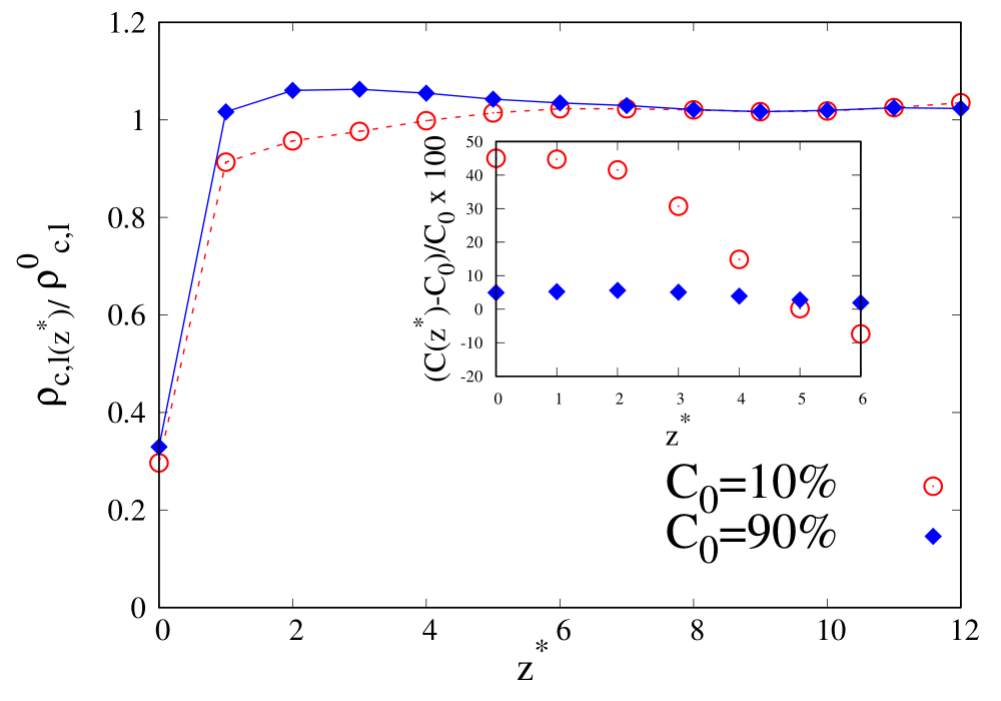
Reduced density profile as a function of the reduced distance from the substrate for 40-mers, wall–bead energy parameter $\epsilon_{w}=1$. Red open circles with dashed line: linear chains at $C_{0}=10\%$; blue full diamonds with full line: cyclic chains at $C_{0}=90\%$. Inset: relative percentage variation in the local composition of cyclic chains; red open circles: $C_{0}=10\%$; blue full diamonds: $C_{0}=90\%$. Lines are a guide and statistical errors are smaller than the symbol size.

Next, we consider a system with 40-mers in Figure 6, and we focus only on the asymmetric compositions ($C_{0}=10\%$, 90%). Upon increasing the chain length of the two species, the density profiles within the ITI (ITI: $z^{*}≲$ 2–9 since $R_{g,b}^{\mathrm{cyc}}\approx 2.1$–2.2 and $R_{g,b}^{\mathrm{lin}}\approx 3$) are compatible with a higher density of cyclic chains compared to linear chains. In fact, the density profile of linear chains is slightly depleted with respect to the bulk density at $C_{0}=10\%$, while the density profile of cyclic chains is slightly enhanced with respect to the bulk value at $C_{0}=90\%$. However, it is especially true for the compositions, which are very different from the equimolar case, that density variations alone cannot be used to understand the quantitatively relative absorption/desorption of one of the two species to the substrate, and that local compositions must be considered.

In this case, the local composition picture reported in the inset of Figure 6 shows a clear enhancement of cyclic chains in the NR for $C_{0}=10\%$ and a nontrivial one of the order of 5% at $C_{0}=90\%$.

**Figure 7:**
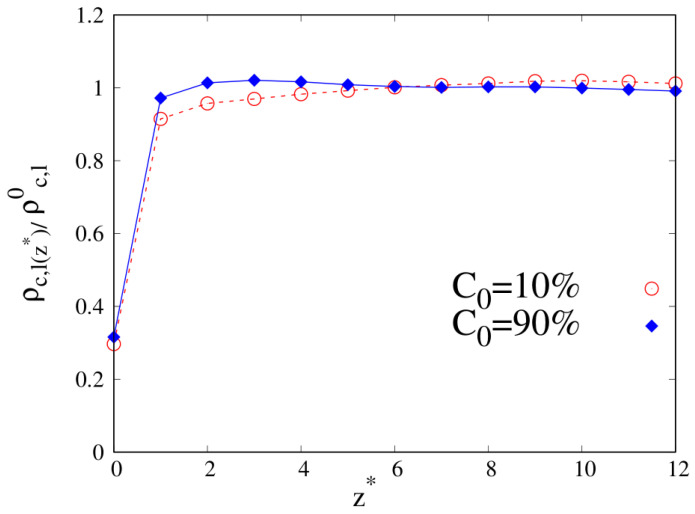
Reduced density profile as a function of the reduced distance from the substrate for 100-mers, wall–bead energy parameter $\epsilon_{w}=1$. Red open circles with dashed line: linear chains at $C_{0}=10\%$; blue full diamonds with full line: cyclic chains at $C_{0}=90\%$. Lines are a guide.

**Figure 8:**
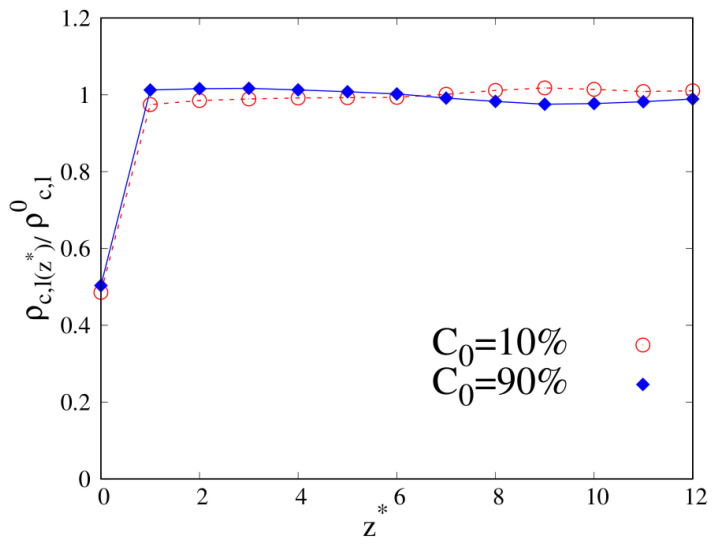
Reduced density profile as a function of the reduced distance from the substrate for 100-mers, wall–bead energy parameter $\epsilon_{w}=2$. Red open circles with dashed line: linear chains at $C_{0}=10\%$; blue full diamonds with full line: cyclic chains at $C_{0}=90\%$. Lines are a guide.

Finally, we focus on the system with the longest chains (100-mers, $z^{*}≲7$–12 since $R_{g,b}^{\mathrm{cyc}}\approx 3.5$ and $R_{g,b}^{\mathrm{lin}}\approx 5$–6) in Figure 7 and Figure 8. The ITI results when the cyclic chains are the minority species still show the same qualitative trend observed for 40-mers at $C_{0}=10\%$, with a slight decrease in the density of linear chains in the SR region; however, the reduced density of linear chains is only marginally above 1 in the opposite regime of composition ($C_{0}=90\%$), as was observed for 40-mers previously.

**Figure 9:**
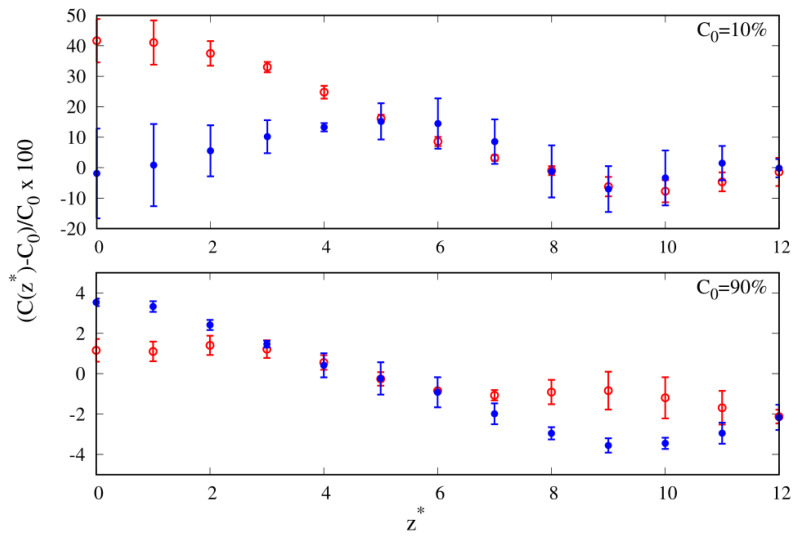
Relative percentage variation in the local composition as a function of the reduced distance from the substrate for 100-mers and three different compositions (see legend). Red color represents energy parameter of the wall–bead attraction $\epsilon_{w}=1$; blue color represents energy parameter of the wall–bead attraction $\epsilon_{w}=2$.

In Figure 9, we show the relative percentage profiles of the local composition as a function of the distance from the wall for $C_{0}=10\%$, 90%. At the lower cyclic composition, we clearly see that cyclic chains become enhanced by as much as 40% in the NR, in agreement with what we previously observed in Figure 6 for 40-mers. At $C_{0}=90\%$, we do not observe any noteworthy local composition variations of at least 5%.

By increasing the wall–polymers energy parameter (attraction) to $\varepsilon_{w}=2$, for $C_{0}=10\%$, we observe the enhancement of the local composition of cyclic chains shifting away from the NR and becoming localized between the NR and SR of the ITI. Finally, at $C_{0}=90\%$, the percentage depletion of cyclic chains becomes barely noticeable (below 5%) and is further shifted into the NR region.

In summary, the analysis of the density profiles indicates that the mixing entropy of the two chains can significantly influence the interfacial free energy of the system and drive clear variations in the adsorption of the two chains at the wall. Since all the van der Waals interactions of the chain beads are identical in our model, we argue that this effect is driven by the different topology of the chains, which affects their local packing constraints in the presence of each other.

We also note that the interfacial behavior observed for 40-mers and 100-mers (long chains) at low cyclic composition ($C_{0}=10\%$) is similar to what has been reported in free-standing films (i.e., forming an interface with empty space) of cyclic and linear polymer chains [17]. In those systems, a preferential adsorption of cyclic chains at the interface was observed across the entire composition range. It was noted that the gap between the cyclic and linear density profiles near the interface tends to shrink with increasing cyclic composition in free-standing films [39]. We argue that, even in the presence of a solid substrate, experiments conducted in the regime of diluted cyclic composition are better suited to detect the preferential adsorption of cyclic chains at the interface.

In the case of 10-mers (short chains), as reported in polymer blends exposed to a vacuum, the relative density indicates the preferential adsorption of linear chains at the interface in the composition range between $C_{0}=10\%$ and 50%. These results present a qualitative picture similar to the numerical and experimental evidence for free-standing films of linear and cyclic polymer blends of comparable chain lengths [40]. Our conclusion is that the enhancement of the local composition for linear polymers should also be observed experimentally for blends exposed to a solid surface in the case of short chains (10-mers).

Even though linear chains have a clear enthalpic advantage in avoiding the wall since they better minimize their energy when surrounded by other chains [41], they are still more enhanced at the interface in the limit of short chains due to their higher flexibility compared to cyclic chains. We showed that linear chains for 10-mers at $C_{0}=50\%$ tend to be even more enhanced when the substrate is made relatively more attractive, a phenomenon that could also be observed experimentally by properly tuning the surface chemistry. Self-consistent field theory predicted that cyclic chains should always be preferentially enhanced at the interface when the chain length becomes sufficiently long [42]. In summary, the picture emerging from our results agrees with theoretical [16] and experimental [11,43] evidence reported in the literature, which describes the enhancement of cyclic chains at the interface for comparably long chain lengths.

It is evident that all the density profiles as a function of the distance from the wall exhibit very limited oscillations, both in number and amplitude, which are a result of layering at the wall. Layering is quite often the result of a competition between attraction at the wall and attractive interactions among fluid particles. The idea is that the substrate must be sufficiently attractive so that the interfacial density grows high enough to begin density oscillations, which eventually fade away from the wall. For hard-sphere fluids, the absence of attraction in the fluid is compensated by the entropic advantage for hard spheres to absorb at the wall; however, for hard-sphere chains, the outcome could be different, or not, because particle bonding may diminish the entropic advantage to enhance wall absorption. For example, hard-sphere chains show layering phenomena when the hard wall is made sticky attractive (see e.g., [44]); however, density oscillations can be depleted if hard sphere chains are exposed to a repulsive wall (see e.g., [45]). When attractive LJ 12-6 interactions are active between particles and the substrate is attractive as well, the outcome depends on the relative magnitude of attractive forces; we also expect that particle bonding versus chain length plays a major role, because the chain topology acts like a rope under tension due to the combined attraction of particles away from the wall. The role of the relative magnitude of particle–particle and particle–wall attraction is evident in the seminal paper by Muller et al. [22], where the same LJ 9-3 wall–fluid potential of our work is used for different values of the attractive depth. Muller et al. demonstrate that if the LJ 9-3 wall potential depth assumes the same value of the LJ 12-6 potential depth, or even twice as much of it (which perfectly fits our choice of the wall energy parameters), density oscillations are considerably damped out because the substrate attraction is not sufficient to produce any wetting of the wall and the formation of a stable film at the interface. Monte Carlo simulation shows that density oscillations in this situation either disappear completely or are detectable as a ripple on a length-scale significantly lower than σ, so they cannot be observed on the scale of Figure 1. Large density oscillations are observed only for higher values of the LJ 9-3 wall depth, as was also reported in [23], or in [46], where layering disappears for a value of the energy parameter in a Steele 10-4-3 potential of less than 2.283 in units of the fluid–fluid attractive potential depth.

### 3.2. Radius of Gyration

In this section, we examine a fundamental shape property of polymer chains by analyzing the average radius of gyration, $R_{g}$, to study the conformation of polymer chains at the polymer/wall interface [47]. The average radius of gyration is defined as follows:

(9) $$R_{g}=\bar{\sqrt{\langle \frac{1}{N_{b}}\sum_{i=1}^{N_{b}}{\left(r_{i}\left(t\right)-r_{\mathrm{com}}\left(t\right)\right)}^{2}\rangle }},$$

where the overline indicates a time average, $N_{b}$ is the total number of beads per polymer, $r_{\mathrm{com}}\left(t\right)$ represents the center-of-mass position of the entire chain, and $r_{i}\left(t\right)$ is the position vector of each bead in the chain at time *t*. The angle brackets 〈·〉 denote an average over all chains within the simulation box.

The parallel ($R_{g}^{\|}$) and perpendicular ($R_{g}^{⊥}$) components of $R_{g}$ are given by

(10) $$R_{g}^{\|}=\bar{\sqrt{\langle \frac{1}{N_{b}}\sum_{i=1}^{N_{b}}\left[{\left(x_{i}\left(t\right)-x_{\mathrm{com}}\left(t\right)\right)}^{2}+{\left(y_{i}\left(t\right)-y_{\mathrm{com}}\left(t\right)\right)}^{2}\right]\rangle }},$$

(11) $$R_{g}^{⊥}=\bar{\sqrt{\langle \frac{1}{N_{b}}\sum_{i=1}^{N_{b}}{\left(z_{i}\left(t\right)-z_{\mathrm{com}}\left(t\right)\right)}^{2}\rangle }}.$$

**Figure 10:**
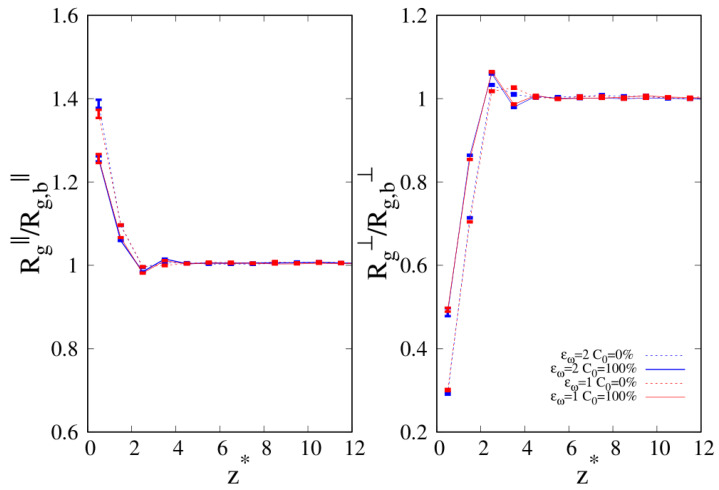
Parallel (**left panel**) and transverse (**right panel**) radius of gyration of pure systems of 10-mers as a function of the reduced distance from the wall. Wall–bead energy parameter $\epsilon_{w}=1$: red lines and error bars; wall–bead energy parameter $\epsilon_{w}=2$: blue lines and error bars. Full lines: pure systems of cyclic chains ($C_{0}=100\%$); dashed lines: pure systems of linear chains ($C_{0}=0\%$). The meaning of the symbols is also reported in the legend and the lines are a guide.

To better understand the density and local composition deviations near the interface, we calculate the three-dimensional 3D radius of gyration $R_{g}$ and its components that are parallel ($R_{g}^{\|}$) and perpendicular ($R_{g}^{⊥}$) to the wall, scaled with respect to their bulk values $R_{g,b}$, as a function of the distance from the wall. In Figure 10, we show $R_{g}^{\|}$ and $R_{g}^{⊥}$ for pure linear and cyclic chains, divided by the corresponding bulk values reported in Table 2 and Table 3.

We can clearly see that upon approaching the substrate, the parallel components of $R_{g}$ tend to increase with respect to their bulk values, with a tendency in the linear chains to achieve higher values of $R_{g}^{\|}$. On the contrary, the vertical component to the substrate of $R_{g}$ tends to shrink with respect to the corresponding bulk value, and as the interface is approached the cyclic $R_{g}^{⊥}$, it becomes fairly smaller than its linear counterpart.

**Figure 11:**
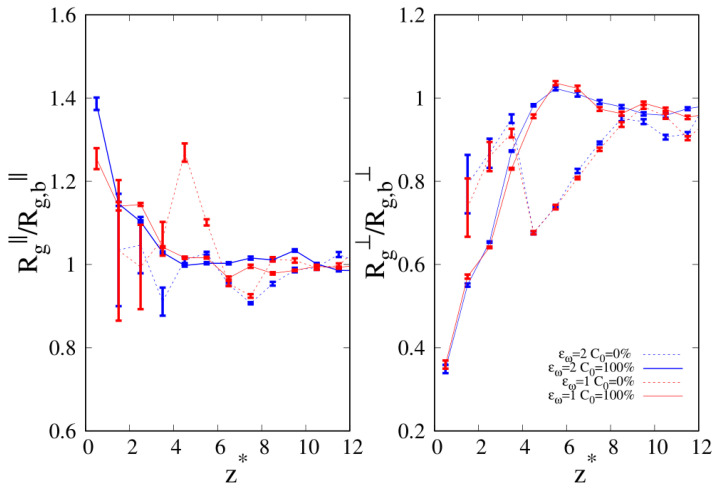
Parallel (**left panel**) and transverse (**right panel**) radius of gyration of pure systems of 100-mers as a function of the reduced distance from the wall. Wall–bead energy parameter $\epsilon_{w}=1$: red lines and error bars; wall–bead energy parameter $\epsilon_{w}=2$: blue lines and error bars. Full lines: pure systems of cyclic chains ($C_{0}=100\%$); dashed lines: pure systems of linear chains ($C_{0}=0\%$). The meaning of symbols is also reported in the legend and the lines are a guide.

When we consider the pure systems of 100-mers in Figure 11, we see that although $R_{g}^{⊥}$ follows the same qualitative behavior observed for 10-mers, the parallel component to the wall $R_{g}^{\|}$ does not. In the latter case, we clearly see the statistical errors becoming particularly large as the interface is approached and even if the linear chains are particularly affected by this problem and we fail to acquire enough statistical data very close to the interface, cyclic chains appear to achieve higher values of $R_{g}^{\|}$ very close to the interface. Even the qualitative behavior of $R_{g}^{\|}$ is affected by the wall–blend attraction because we note that the amplitude of the oscillation of $R_{g}^{\|}$ which develops for a mild attraction to the substrate ($\epsilon_{w}=1$, see the red, dashed line of the left panel) is significantly smoothed in the case of stronger attraction to the substrate ($\epsilon_{w}=2$).

**Figure 12:**
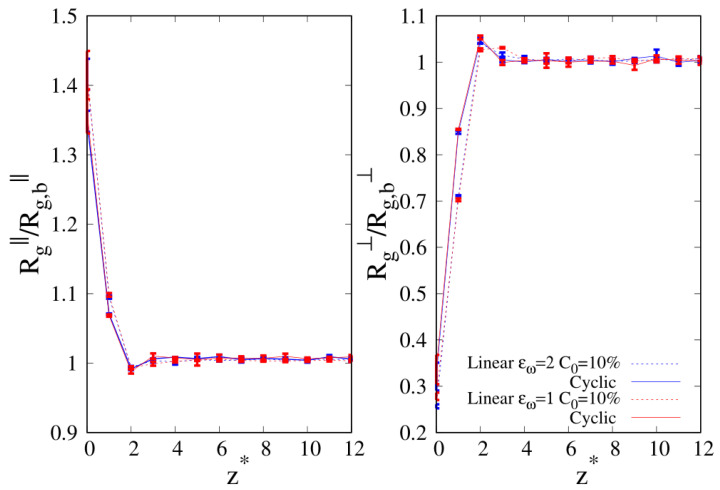
$C_{0}=10\%$. Parallel (**left panel**) and transverse (**right panel**) radius of gyration of 10-mers blends as a function of the reduced distance from the wall. Wall–bead energy parameter $\epsilon_{w}=1$: red lines and error bars; wall–bead energy parameter $\epsilon_{w}=2$: blue lines and error bars. Full lines: cyclic chains; dashed lines: linear chains. The meaning of symbols is reported in the legend and the lines are a guide.

In Figure 12, we show the same quantities for 10-mers at $C_{0}=10\%$ and wall attraction $\epsilon_{w}=1,2$, where we found evidence for the enhancement of the local composition of linear chains. Within the large error bars close to the interface, we observe a larger increase in the linear $R_{g}^{\|}$ in the NR, signaling a slightly more pronounced tendency of linear chains to adopt a shape parallel to the wall, regardless of the wall affinity, i.e., for both $\varepsilon_{w}=1$ and $\varepsilon_{w}=2$. When examining $R_{g}^{⊥}$, the opposite trend is observed: cyclic chains appear more oriented perpendicularly to the wall. However, while $R_{g}^{\|}$ increases to values well above its bulk value $R_{g,b}^{\|}$ (the ratio reported in Figure 11 is greater than one), $R_{g}^{⊥}$ decreases to values well below $R_{g,b}^{⊥}$.

It appears that in a regime where cyclic chains are the minority component, linear chains gain a higher amount of conformational entropy by adopting a more swollen shape along the direction parallel to the interface, similarly to what has been observed in systems of pure cyclic chains [48], where topological excluded volume interactions play a role. The average value (middle point of the error bar) of $R_{g}^{\|}$ for linear chains at mild substrate attraction ($\varepsilon_{w}=1$) is only slightly higher than at stronger attraction ($\varepsilon_{w}=2$), for which we do not observe any significant enhancement of linear chains in Figure 5. This suggests that the final outcome at the interface results from a delicate balance between the entropy gain/loss associated with the two components of the radius of gyration.

However, conformational entropy is not the only entropic contribution favoring cyclic chains at the interface. It is well known that linear chains have additional mechanisms to increase their entropy at the interface (and minimize the free energy), especially when cyclic chains are the majority component [49]. The exposure of their chain ends at the interface allows linear chains to minimize entropy loss since only one bond emerges from the end segment.

Self-Consistent Field Theory (SCF) [19], for sufficiently small concentrations of cyclic chains, predicts that the density profile of linear chains at the interface is produced by attractive surface potentials of entropic origin, which arise due to the exposure of their chain ends. This phenomenon has also been observed in polystyrene blends using neutron reflectometry and Raman spectroscopy experiments [3], and it has been confirmed numerically in molecular dynamics simulations of chains with linear and cyclic topology [50]. The density of end segments in bead–spring models of homopolymer systems of linear chains has also been found to be enhanced in Monte Carlo simulations [7,51]. Of course, the behavior observed in Figure 12 has its origin in the picture that we discussed in Figure 10 for the pure systems of 10-mers.

**Figure 13:**
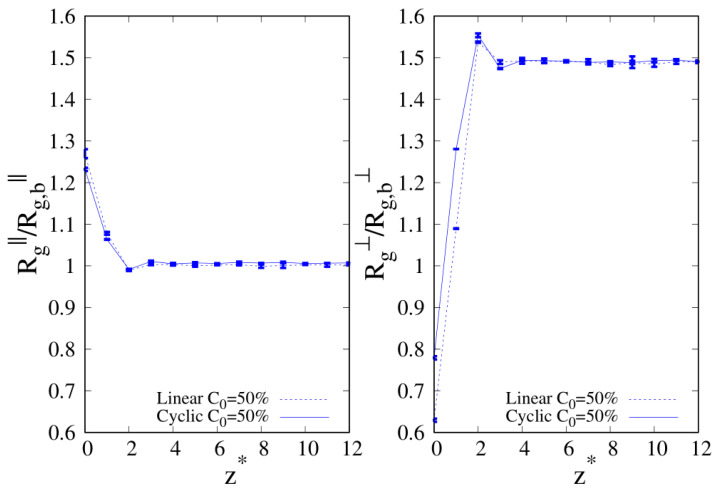
$C_{0}=50\%$ and wall–bead energy parameter $\epsilon_{w}=2$. Parallel (**left panel**) and transverse (**right panel**) radius of gyration of 10-mers blends as a function of the reduced distance from the wall. Full lines: cyclic chains; dashed lines: linear chains. The meaning of the symbols is also reported in the legend and the lines are a guide.

In Figure 13, we consider the equimolar case $C_{0}=50\%$ at stronger wall attraction ($\varepsilon_{w}=2$), where we found an enhancement of the local composition of linear chains in the NR of the ITI. We again observe the same qualitative scenario reported for the pure systems and for the case of $C_{0}=10\%$, where linear chains are the majority species (see Figure 10, Figure 11 and Figure 12), confirming our impression that the real entropy gain/loss originates from a subtle competition between the parallel and perpendicular components of the radius of gyration.

**Figure 14:**
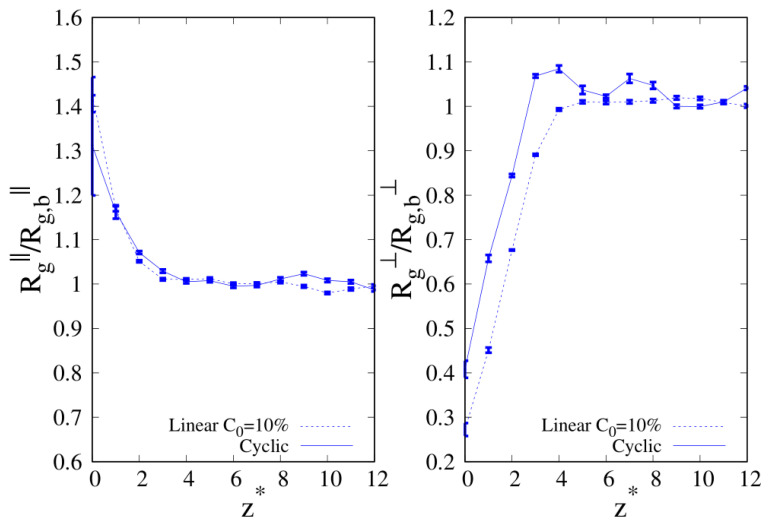
$C_{0}=10\%$ and wall–bead energy parameter $\epsilon_{w}=1$. Parallel (**left panel**) and transverse (**right panel**) radius of gyration of 40-mers blends as a function of the reduced distance from the wall. Full lines: cyclic chains; dashed lines: linear chains. The meaning of the symbols is also reported in the legend and the lines are a guide.

We report the two components of $R_{g}$ in Figure 14 for the case $C_{0}=10\%$ at stronger wall affinity ($\varepsilon_{w}=2$), where we observed an enhancement of cyclic chains at the wall. If we look at the parallel component of $R_{g}$ on the left panel and we move along the z axis towards the wall, we clearly see that we enter the ITI with the parallel component of cyclic $R_{g}$ above one of the linear chains, and as we move closer to the wall, even if the linear $R_{g}^{\|}$ appears above the cyclic one, the large error bars prevent us from making a definite conclusion about which of the two chains is gaining more entropy. However, the results for the perpendicular component to the wall $R_{g}^{⊥}$ corroborate the idea that cyclic chains lose less conformational entropy than linear chains, supporting the evidence reported in Figure 6 of the enhancement of the local composition of cyclic chains at the interface. The increase in the chain length brings us in a new territory, and while the $R_{g}^{⊥}$ is always larger for cyclic chains as the interface is approached, $R_{g}^{\|}$ stops showing a neat separation between the two different chains (as was the case for shorter chain lengths), signaling that the linear chains are not gaining more conformational entropy than the cyclic chains.

**Figure 15:**
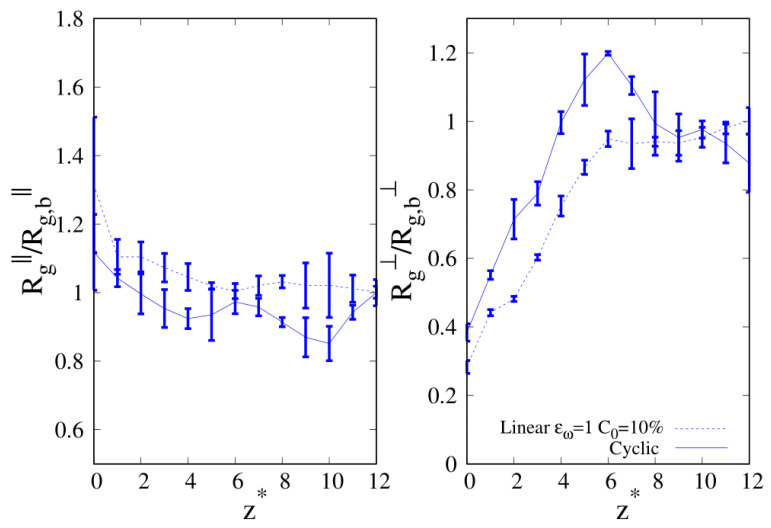
Parallel (**left panel**) and transverse (**right panel**) radius of gyration of 100-mers blends as a function of the reduced distance from the wall. Wall–bead energy parameter $\epsilon_{w}=1$ and $C_{0}=10\%$: blue lines and error bars. The meaning of the symbols is also reported in the legend and the lines are a guide.

Finally, in Figure 15, we show the results for the radius of gyration of 100-mers at the composition where we observed cyclic ($C_{0}=0.1$, $\varepsilon_{w}=1$). Due to the large error bars, these results appear inconclusive for the parallel component of $R_{g}$ (see left panel), but as it was shown before for 40-mers for $C_{0}=0.1$, $\varepsilon_{w}=1$, the perpendicular component $R_{g}^{⊥}$ of the cyclic chains assumes the largest values in the NR, suggesting that the cyclic enhancement at the interface might be driven by the comparatively minor loss of conformational entropy with respect to linear chains as the interface is approached.

### 3.3. Energy

**Figure 16:**
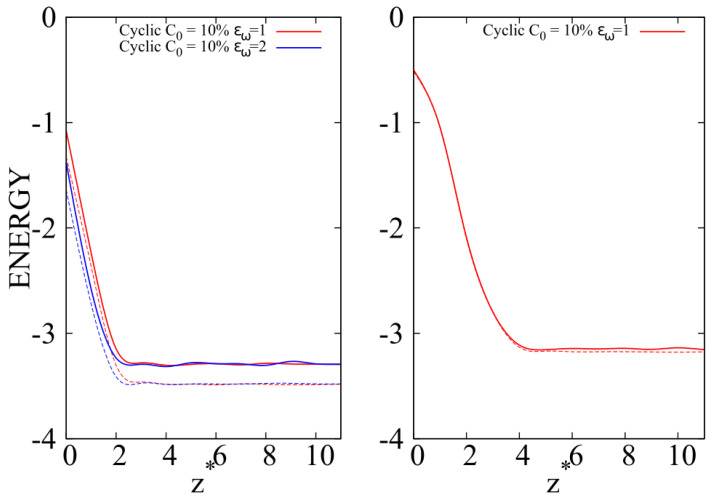
Energy per bead as a function of the reduced distance from the substrate. Full lines: cyclic chains; dashed lines with the same color of full lines: linear chains at the corresponding composition reported in the legend. In the legend, we do not report the dashed lines (linear chains). Left panel: 10-mers; red full line: linear chains at $\epsilon_{w}=1$, $C_{0}=10\%$; red dashed line: cyclic chains at $\epsilon_{w}=1$, $C_{0}=10\%$; blue full line: linear chains at $\epsilon_{w}=2$, $C_{0}=10\%$; blue dashed line: cyclic chains at $\epsilon_{w}=2$, $C_{0}=10\%$; Right panel: 100-mers; red full line: linear chains at $\epsilon_{w}=1$, $C_{0}=10\%$; red dashed line: cyclic chains at $\epsilon_{w}=1$, $C_{0}=10\%$; The meaning of the symbols is also reported in the legend and the lines are a guide.

In the case of 10-mers and in the ITI, at $C_{0}=10\%$ and mild wall attraction $\varepsilon_{w}=1$, the linear chains are energetically favored in comparison to cyclic chains, as reported in the left panel of Figure 16, where we show the energy per bead of the two different chains as a function of the distance from the wall, excluding the interaction energy with the wall. By increasing the wall attraction to $\varepsilon_{w}=2$, we observe that the energy difference between cyclic and linear chains is very similar, with the absolute values being lower due to the higher value of the energy parameter for the wall–bead interaction. Linear enhancement at the wall, as shown in Figure 5, appears to be driven by the higher flexibility of short linear chains compared to their cyclic counterparts, which allows them to better minimize the interfacial free energy by maximizing the number of energy contacts with surrounding beads.

Energy does not seem to play a significant role when the chain length becomes sufficiently long, as shown in the right panel of Figure 14, where we report the energies per bead as a function of the distance from the wall for 100-mers for the case where we observed cyclic enhancement at $C_{0}=10\%$, $\varepsilon_{w}=1$ (Figure 9, top panel). In the case of longer chain lengths, this result is expected because the loop geometry becomes less relevant in constraining a cyclic chain in a smaller volume, allowing its beads to interact efficiently with their local environment in a not too dissimilar way than the ones of the linear chains.

### 3.4. Diffusion Coefficients

In this section, we analyze the diffusion coefficient of the polymer chains, which can be estimated in terms of the slope of the mean square displacement (MSD) of the center-of-mass of polymer molecules in different slabs of thickness σ along the z-direction of the simulation box, using Einstein’s relation:

(12) $$\langle |{\bar{\Delta R}}_{com}{|}^{2}\rangle =2nD\Delta t$$

where *D* is the diffusion coefficient of the chains in the mixture, $\langle |{\bar{\Delta R}}_{com}{|}^{2}\rangle$ is the mean square displacement vector of their center of mass, Δt is the time over which the displacement occurs, and *n* is the allowed dimensionality of the diffusion process. Since the presence of the surface breaks down the symmetry of the diffusion of the polymer chains in the directions parallel and perpendicular to the surface, it is customary to break down the diffusion coefficient into its parallel ($D^{\|}$, n=2) and perpendicular ($D^{⊥}$, n=1) components. Here, the components are scaled by their respective bulk values, which are denoted by $D_{0}^{\|}$ and $D_{0}^{⊥}$, respectively, and are reported in Table 4 and Table 5 for different chains lengths, compositions, and wall energy parameters.

**Figure 17:**
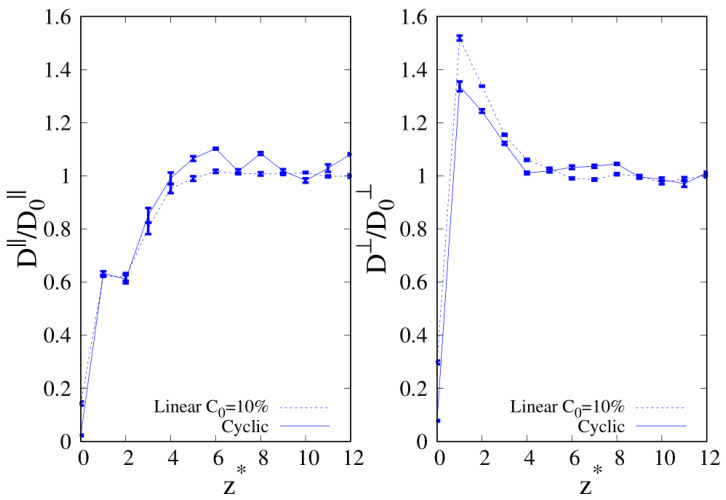
$C_{0}=10\%$ and wall–bead energy parameter $\epsilon_{w}=1$. Parallel (**left panel**) and transverse (**right panel**) diffusion coefficients of 10-mers blends as a function of the reduced distance from the wall. Full lines: cyclic chains; dashed lines: linear chains. The meaning of the symbols is also reported in the legend and the lines are a guide.

**Figure 18:**
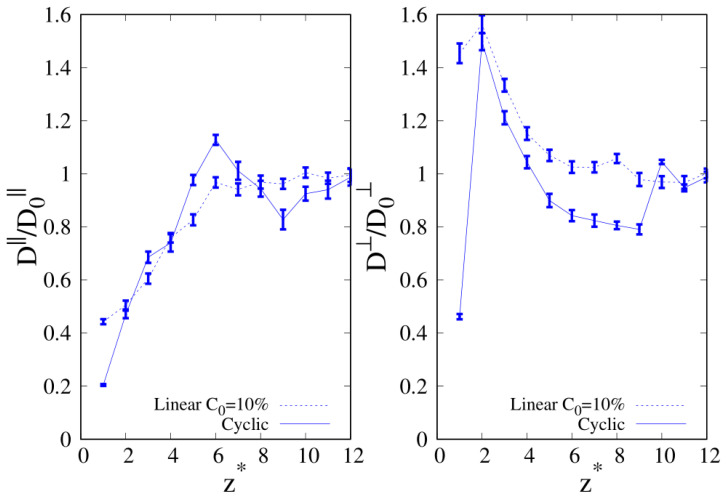
$C_{0}=10\%$ and wall–bead energy parameter $\epsilon_{w}=1$. Parallel (**left panel**) and transverse (**right panel**) diffusion coefficients of 100-mers blends as a function of the reduced distance from the wall. Full lines: cyclic chains; dashed lines: linear chains. The meaning of the symbols is also reported in the legend and the lines are a guide.

The results for diffusivity as a function of the distance from the substrate are reported in Figure 17 and Figure 18 for the selected cases where we found an increased relative absorption of linear (10-mers) and cyclic (100-mers) chains in the regime where linear chains are the majority component (as guided by experiments for polymer blends exposed to vacuum [11,16]). The lack of significant statistical data for the MSD within σ from the substrate for 100-mers prevented us from obtaining an estimate of the components of *D* that was very close to the interface. We found that for both the parallel and transverse components of the diffusion coefficient, their values drop significantly as the interface is approached, which is expected for the chain beads that are very close to the solid substrate. In fact, the diffusion coefficient is observed to increase monotonically for free standing polymer chains, which are exposed to empty space [39]. However, the behavior observed for the transverse component is different than the one of the parallel component, since we observed that the diffusion in the normal direction to the substrate tends to increase as the interface is approached, and it drops drammatically only when the beads are very close to the substrate (see right panels of Figure 17 and Figure 18). The mobility of linear chains along the parallel direction to the substrate is only marginally lower than the one of cyclic chains, and this observation is reversed for the mobility in the transverse direction to the substrate. However, in the case of short chains (see the right panel of Figure 17), the maximum of mobility is nearly 20% higher for linear chains, which can be seen as a factor contributing to the enhancement of the composition of linear polymers at the interface. The observed suppression of mobility at close range with the substrate is coherent with the experimental findings, which report a dramatic drop of surface dynamics below one radius of gyration from the interface [34].

## 4. Conclusions

In this work, we have demonstrated that blending polymer chains of distinct topologies, specifically linear and cyclic chains, leads to nontrivial structural effects at interfaces, which cannot be readily inferred from their individual adsorption behaviors.

Our simulations provide numerical evidence that the local enrichment of short linear chains at the interface remains robust, even in the presence of an attractive substrate. This behavior is primarily driven by their greater conformational flexibility in comparison to cyclic chains, which enhances their conformational entropy and provides an enthalpic advantage through increased interactions with surrounding chains.

In contrast, for longer chains, we find that cyclic chains are preferentially adsorbed at the interface, in agreement with previous findings on free-standing films composed of linear and cyclic polymer blends. However, we hypothesize that this trend may be altered in the presence of high-energy surfaces, where the intrinsic flexibility of linear chains may continue to favor their localization at the interface.

Given the complexity and often elusive nature of the behavior of polymers in nanometer-thick films, this study represents an initial step toward understanding the molecular origins of confinement effects and the influence of chain topology on the structure and thickness of the adsorbed layer.

Future work will aim to explore these findings further, particularly in relation to the dynamic properties of the system and how they are influenced by, or coupled to, the distinct topologies of linear and cyclic chains near solid interfaces. We hope to address these questions in a subsequent study.

## Figures and Tables

**Table 1:** Bulk average densities for cyclic ($\rho_{C}$) and linear ($\rho_{L}$) chains in $\sigma^{3}$ units.

$N_{b}$ $C_{0}$	$\rho_{C}$	$\rho_{L}$
10:0%	−	0.87
10:10%	0.87	0.78
10:50%	0.44	0.44
10:90%	0.80	0.09
10:100%	0.89	−
40:10%	0.09	0.79
40:90%	0.80	0.09
100:0%	−	0.89
100:10%	0.09	0.80
100:50%	0.44	0.44
100:90%	0.80	0.09
100:100%	0.89	−

**Table 2:** Bulk radius of gyration ($R_{g,b}$), parallel ($R_{g,b}^{\|}$), and perpendicular ($R_{g,b}^{⊥}$) components to the substrate at wall–bead energy parameter $\epsilon_{w}=1$.

% Cyclic Concentration	100-mers		40-mers		10-mers	
	Linear($R_{g,b}$):($R_{g,b}^{\|}$):($R_{g,b}^{⊥}$)	Cyclic($R_{g,b}$):($R_{g,b}^{\|}$):($R_{g,b}^{⊥}$)	Linear($R_{g,b}$):($R_{g,b}^{\|}$):($R_{g,b}^{⊥}$)	Cyclic($R_{g,b}$):($R_{g,b}^{\|}$):($R_{g,b}^{⊥}$)	Linear($R_{g,b}$):($R_{g,b}^{\|}$):($R_{g,b}^{⊥}$)	Cyclic($R_{g,b}$):($R_{g,b}^{\|}$):($R_{g,b}^{⊥}$)
10	5.1(1):3.54(1):2.8(1)	3.8(2):2.97(1):2.1(2)	3.13(8):2.52(4):1.7(1)	2.36(1):1.91(1):1.33(5)	1.45:2.05(2):0.79(1)	1.14:0.92:0.64
50	5.3(1):4.06(6):2.8(1)	3.60(4):2.92(2):2.02(5)			1.45:1.16:0.79(2)	1.14:0.92:0.64
90	6.0(4):4.24(2):2.8(3)	3.51(3):2.84(2):1.97(4)	3.07:4.34(3):1.68(4)	2.29:1.82(1):1.29(2)	1.49(1):1.16(2):0.78(1)	1.14:0.92(1):0.64

**Table 3:** Bulk radius of gyration ($R_{g,b}$), parallel ($R_{g,b}^{\|}$), and perpendicular ($R_{g,b}^{⊥}$) components in the substrate at wall–bead energy parameter $\epsilon_{w}=2$.

% Cyclic Concentration	100-mers		10-mers	
	Linear($R_{g,b}$):($R_{g,b}^{\|}$):($R_{g,b}^{⊥}$)	Cyclic($R_{g,b}$):($R_{g,b}^{\|}$):($R_{g,b}^{⊥}$)	Linear($R_{g,b}$):($R_{g,b}^{\|}$):($R_{g,b}^{⊥}$)	Cyclic($R_{g,b}$):($R_{g,b}^{\|}$):($R_{g,b}^{⊥}$)
10	5.2(1):4.07(6):2.80(9)	3.8(1):3.05(7):2.1(1)	1.45:1.16:0.79	1.13(7):0.92(4):0.63(4)
50	5.1(2):4.24(2):2.8(2)	3.62(6):2.94(4):2.03(8)	1.44:1.16:0.79	1.14:0.92:0.64
90	6.0(5):4.24(2):2.8(2)	3.51(3):2.86(2):1.96(3)	1.45:1.16:0.79	1.14:0.92:0.64

**Table 4:** Parallel ($D^{\|}$) and perpendicular ($D^{⊥}$) components for the diffusion coefficient for wall–bead energy parameter $\epsilon_{w}=1$. Unless explicitly reported, errors are on the last digit.

% Cyclic Concentration	100-mers		40-mers		10-mers	
	Linear($D^{\|}$):($D^{⊥}$)	Cyclic($D^{\|}$):($D^{⊥}$)	Linear($D^{\|}$):($D^{⊥}$)	Cyclic($D^{\|}$):($D^{⊥}$)	Linear:($D^{\|}$):($D^{⊥}$)	Cyclic:($D^{\|}$):($D^{⊥}$)
10	0.02 : 0.02	0.01 : 0.01	0.05 : 0.05	0.03 : 0.03	0.20 : 0.20	0.10 : 0.11
90	0.05 : 0.06	0.01 : 0.01	0.11 : 0.09(2)	0.03 : 0.03	0.20 : 0.21	0.10 : 0.10

**Table 5:** Parallel ($D^{\|}$), and perpendicular ($D^{⊥}$) components of the diffusion coefficient for $\epsilon_{w}=2$. Unless explicitly reported, errors are on the last digit.

% Cyclic Concentration	100-mers		10-mers	
	Linear($D^{\|}$):($D^{⊥}$)	Cyclic($D^{\|}$):($D^{⊥}$)	Linear($D^{\|}$):($D^{⊥}$)	Cyclic($D^{\|}$):($D^{⊥}$)
10	0.02 : 0.02	0.01 : 0.01	0.20 : 0.20	0.11 : 0.11
90	0.08 : 0.06(2)	0.01 : 0.01	0.20 : 0.20	0.10 : 0.10

## Data Availability

Data are contained within the article.
